# Endocrinologically Silent Crooke Cell Adenoma With Apoplexy Causing Oculomotor Palsy: An Uncommon and Underrecognised Entity

**DOI:** 10.7759/cureus.115044

**Published:** 2026-08-23

**Authors:** Ernest Zhi Yu Tay, Vint Seng Hein Lin Htut Lee, Char Loo Tan, Bing Cheng Wu, Ira Siyang Sun, Shiong Wen Low, Chun Peng Goh

**Affiliations:** 1 Department of Neurosurgery, Ng Teng Fong General Hospital, Singapore, SGP; 2 Department of Pathology, National University Health System, Singapore, SGP

**Keywords:** corticotroph, crooke cell adenoma, endocrinologically silent, pitnet, pituitary apoplexy, pituitary neuroendocrine tumour, transsphenoidal surgery

## Abstract

Crooke cell adenomas (CCAs) are among the most aggressive pituitary neuroendocrine tumours (PitNET). The convergence with pituitary apoplexy and endocrinological silence is very rare, as these factors add to the diagnostic complexity and delayed recognition of this entity. A 41-year-old male patient with a known untreated non-functional pituitary macroadenoma, presented with acute right oculomotor palsy and headache. Magnetic resonance imaging (MRI) demonstrated haemorrhagic infarction with bilateral internal carotid artery (ICA) encasement and cavernous sinus extension. He underwent urgent endoscopic endonasal transsphenoidal resection. Subtotal resection was performed given bilateral cavernous ICA encasement. Post-operatively, there was complete resolution of the oculomotor palsy and he remained endocrinologically silent throughout. Given its aggressive histological profile, adjuvant volumetric modulated arc therapy (VMAT, 54 Gy in 30 fractions) was administered three months postoperatively, with stable residual disease on serial MRI. This case is the tenth documented case of CCA presenting with pituitary apoplexy. Its paucity in the literature reflects not only the rarity of CCA with apoplexy but also underdiagnosis driven by its silent clinical presentation, absence of standardised histological protocols for apoplectic specimens, and immunohistochemical constraints in necrosis-heavy specimens. This case is the first to document subtotal resection with adjuvant fractionated radiotherapy as a safe and effective strategy in CCA apoplexy complicated by ICA encasement. CCA must be actively excluded in corticotroph pituitary neuroendocrine tumours (PitNETs) presenting with apoplexy, with viable tissue undergoing a minimum staining panel. Endocrinological silence can be falsely reassuring, as tumour aggressiveness is predicted by morphology and molecular profile rather than cortisol levels. Post-treatment surveillance via neuroimaging is crucial in biochemically silent CCA. Multicentre collaboration and prospective registry data are essential to establish evidence-based protocols for this rare but clinically consequential entity.

## Introduction

The 2022 World Health Organization (WHO) classification redefined pituitary adenomas as pituitary neuroendocrine tumours (PitNETs), reflecting a shift towards lineage-based classification by transcription factors and immunohistochemistry [1]. Among T-box transcription factor (TPIT)-driven corticotroph tumours, Crooke cell adenomas (CCAs) are one of the most aggressive [2], presenting as macroadenomas in 81% of cases with high rates of postoperative recurrence (56%) and invasive behaviour (71-81%) [3,4]. Despite this, CCAs account for fewer than 1% of all PitNETs [2].

Pathologically, CCAs demonstrate strong TPIT reactivity with Crooke cell change (perinuclear deposition of hyaline cytokeratin filaments [2,5] in more than 50% of tumour cells [2]). These adenomas often display positive adrenocorticotropic hormone (ACTH), cytokeratin 8/18 (CK8/18) and CAM5.2 immunohistochemistry, with variable periodic acid-Schiff (PAS) and anti-cytokeratin 1/3 (AE1/AE3) expression [2,3,5]. This staining pattern is in response to glucocorticoid excess [4]. CCAs also show a higher prevalence of tumour protein 53 (TP53) and fewer ubiquitin-specific peptidase 8 (USP8) mutations than other corticotroph tumours [6,7]. Radiologically, CCAs are larger than non-CCA corticotroph tumours and predominantly invade the suprasellar region (64%) and cavernous sinus (46%) [3,4].

CCAs typically present in middle-aged adults with female predominance (75%) at a mean age of 46 years. Approximately 65% cause overt Cushing disease; the remaining 35% are endocrinologically silent (positive corticotroph histology without clinical or biochemical evidence of hypercortisolism). This silent subset is particularly difficult to identify preoperatively [4].

Pituitary apoplexy results from haemorrhagic infarction within a pituitary tumour, complicating 2-12% of pituitary adenomas [8]. Patients often present with sudden severe headache, visual disturbances, ophthalmoplegia, and potentially life-threatening adrenal insufficiency [9-11]. Apoplexy is rare in CCAs with only nine documented cases in literature, of which only one presented with severe Cushing’s disease [11-16]. No standardised radiotherapy or chemotherapy protocols specific to CCA currently exist. Evidence on CCA apoplexy management is even more scarce.

This report presents the tenth documented case of CCA presenting with pituitary apoplexy, and the first to document the efficacy of adjuvant volumetric modulated arc therapy (VMAT). It aims to characterise the diagnostic barriers underpinning the underrecognition of CCA apoplexy and to propose a multimodal management framework.

## Case presentation

A 41-year-old male patient with no significant past medical history was referred with an untreated pituitary macroadenoma, first identified in July 2025 following five months of intermittent headaches and facial pain. Magnetic resonance imaging (MRI) demonstrated a 2.8×2.0×1.8 cm sellar mass with right cavernous sinus extension and encasement of the right internal carotid artery (ICA). He subsequently defaulted all Ophthalmology, Endocrinology and Neurosurgery follow-ups.

In January 2026, he re-presented with three days of worsening right-sided headache, periorbital pain, and diplopia. Physical examination revealed a partial right oculomotor nerve palsy with ptosis, anisocoria (3 mm reactive right, 2 mm reactive left), and preserved extraocular movements. Pituitary hormone screen revealed central hypogonadism, raised ACTH and raised 8 a.m. cortisol, the latter confounded by dexamethasone use (Table 1). No Cushingoid features were present. Endocrinology attributed his elevated ACTH levels to stress-related suppression failure rather than autonomous secretion.

**Table 1:** Laboratory results for the patient with Crooke cell adenoma apoplexy (pre-operative, post-operative day 6, one month and three months)

Hormone	Reference Range and Units	Pre-operative	Post-operative Day 6	Post-operative 1 month	Post-operative 3 months
Adrenocorticotropic hormone (ACTH)	1.6-13.9 pmol/L	26.8 (High)	-	21.4 (High)	14.3 (High)
8 a.m. Cortisol	101-536 nmol/L	616	248	29	157
Synacthen test (cortisol)	nmol/L		-	276 (0 min) 613 (30 min) 714 (60 min)	-
Insulin growth factor 1 (IGF1)	96-226 ug/L	142	-	-	-
Prolactin	73-407 mIU/L	303	-	-	-
Follicle-stimulating hormone (FSH)	≤11.9 IU/L	2.4	-	-	-
Luteinizing hormone (LH)	0.6-12.0 IU/L	2.0	-	-	-
Testosterone	8.3-30.2 nmol/L	2.9	-	-	-
Thyroid Stimulating Hormone (TSH)	0.35-4.94 mIU/L	2.35	-	-	-
Free Thyroxine (fT4)	9-19.1 pmol/L	11.5	-	-	-

**Figure 1:**
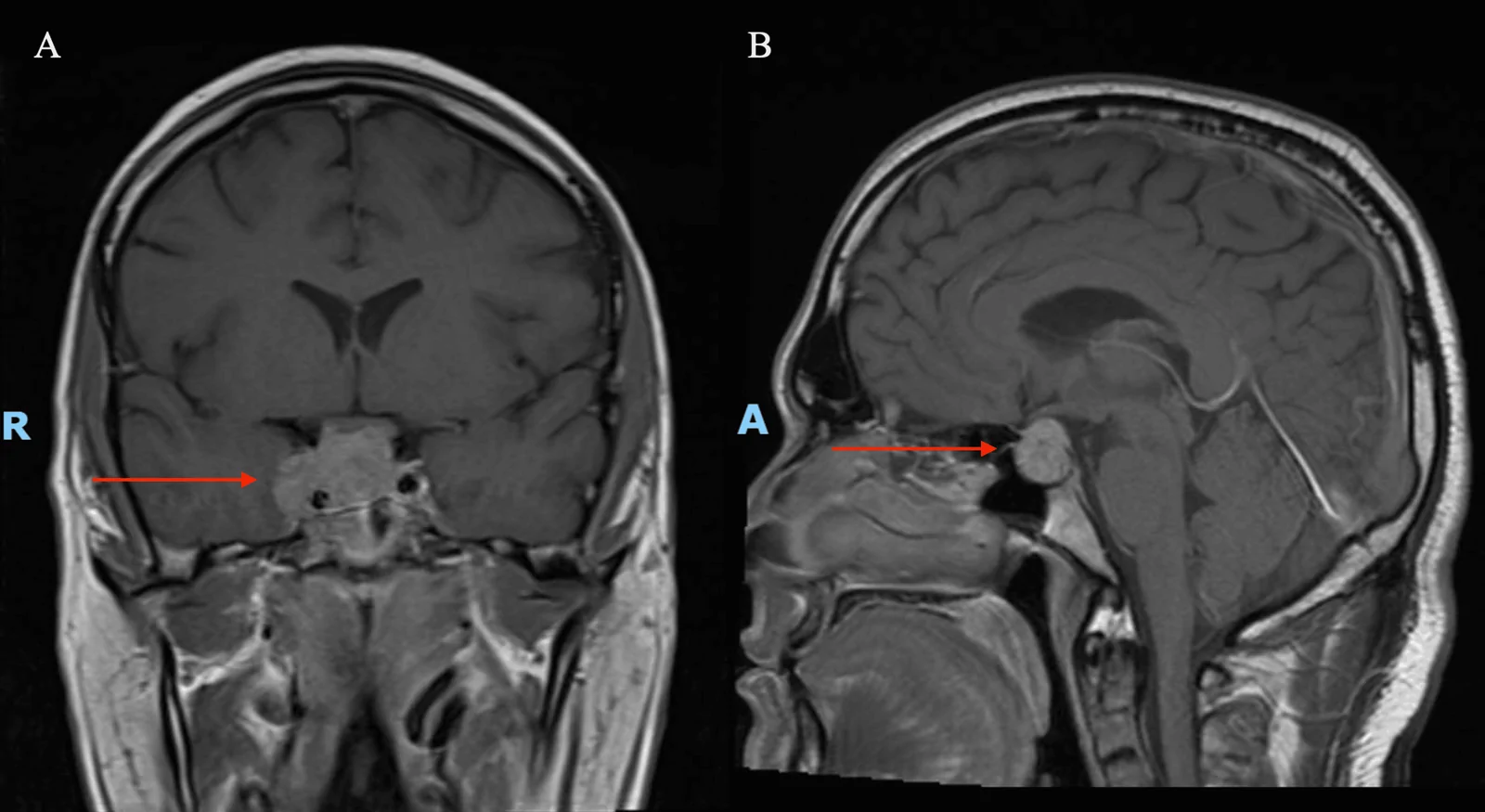
Preoperative images (T1-weighted with contrast coronal (A), sagittal (B)) showing a mildly enhancing pituitary macroadenoma approximately 2.8x2.0x1.8 cm extending into the right cavernous sinus encasing the right cavernous internal carotid artery and slightly into the left cavernous sinus with abutment of the left cavernous internal carotid artery. There is also suprasellar extension with slight lifting of the optic chiasm.

**Figure 2:**
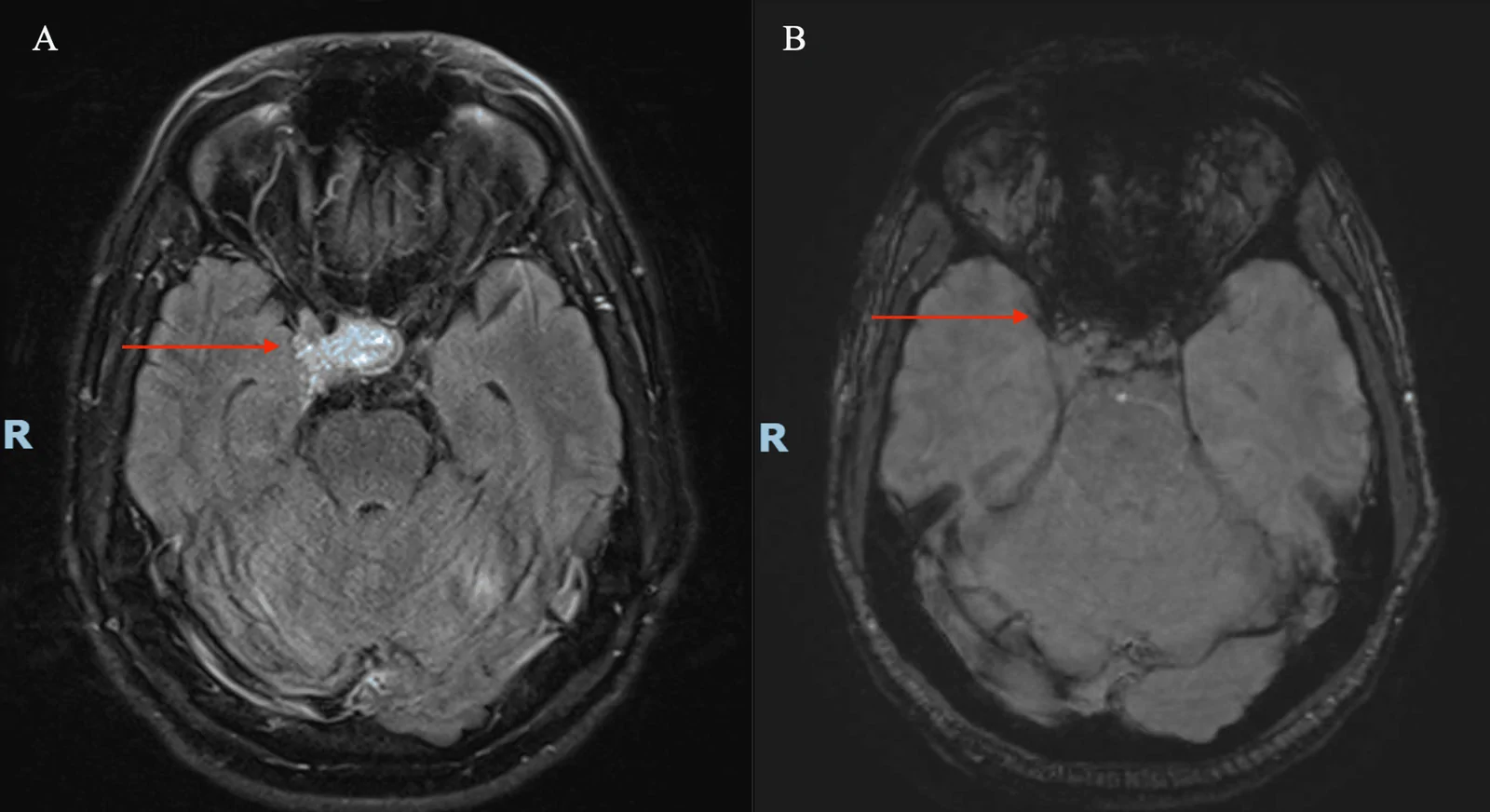
Pituitary macroadenoma visualised with fluid-level seen on T2 weighted fluid-attenuated inversion recovery (FLAIR) axial cuts (A), and blooming effect in the sellar region on susceptibility-weighted imaging (SWI) sequence (B), suggestive of pituitary apoplexy.

MRI of the brain revealed a similarly sized pituitary macroadenoma with new mild superior displacement of the optic chiasm, abutting the left ICA and extending into the right cavernous sinus (Figure 1). There was also an intrasellar fluid level consistent with pituitary apoplexy (Figure 2).

**Figure 3:**
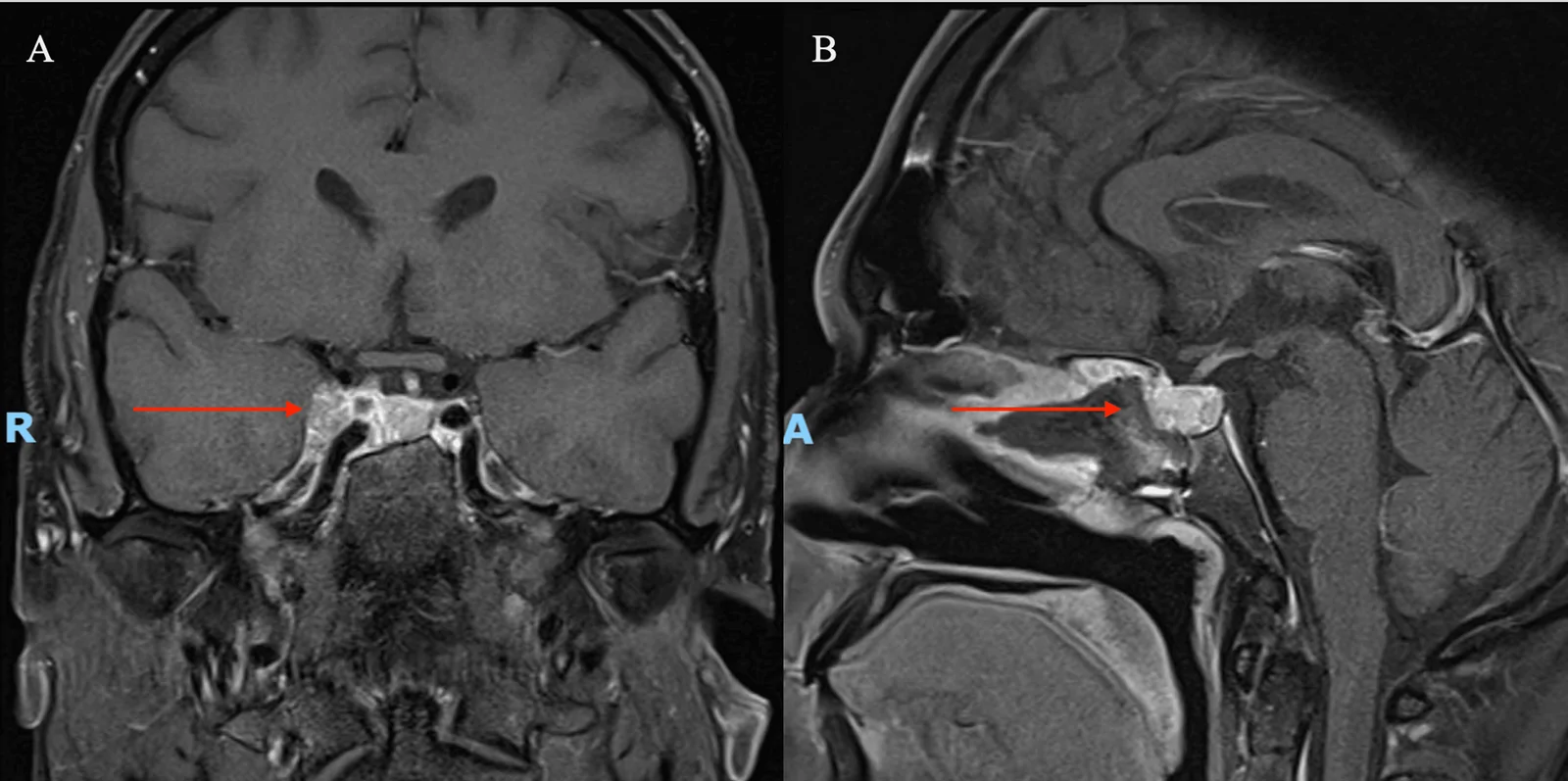
Postoperative images (T1-weighted with contrast coronal (A), sagittal (B)) showing residual tumour lateral to the right internal carotid artery, with reduced suprasellar extension and relieved mass effect on the optic chiasm.

The following day, the patient developed a complete right oculomotor palsy with anisocoria (5 mm right, 3 mm reactive left). Baseline visual acuity was 6/6 bilaterally; this deteriorated to 6/12 in the right eye on admission. He underwent urgent endoscopic endonasal transsphenoidal resection. A right middle turbinectomy, bilateral sphenoidotomies, sellotomy, and curvilinear durotomy were performed, with careful debulking from inferior to lateral via meticulous extracapsular dissection. Subtotal resection (STR) was performed. Tumour extending to the superoposterior margin of the right cavernous sinus was intentionally left in-situ to avoid vascular injury. Diaphragmatic descent was visualised intraoperatively with postoperative MRI confirming good decompression of the chiasm (Figure 3). The sellar defect was reconstructed with a multilayer closure technique. There was no intraoperative cerebrospinal fluid (CSF) leak.

**Figure 4:**
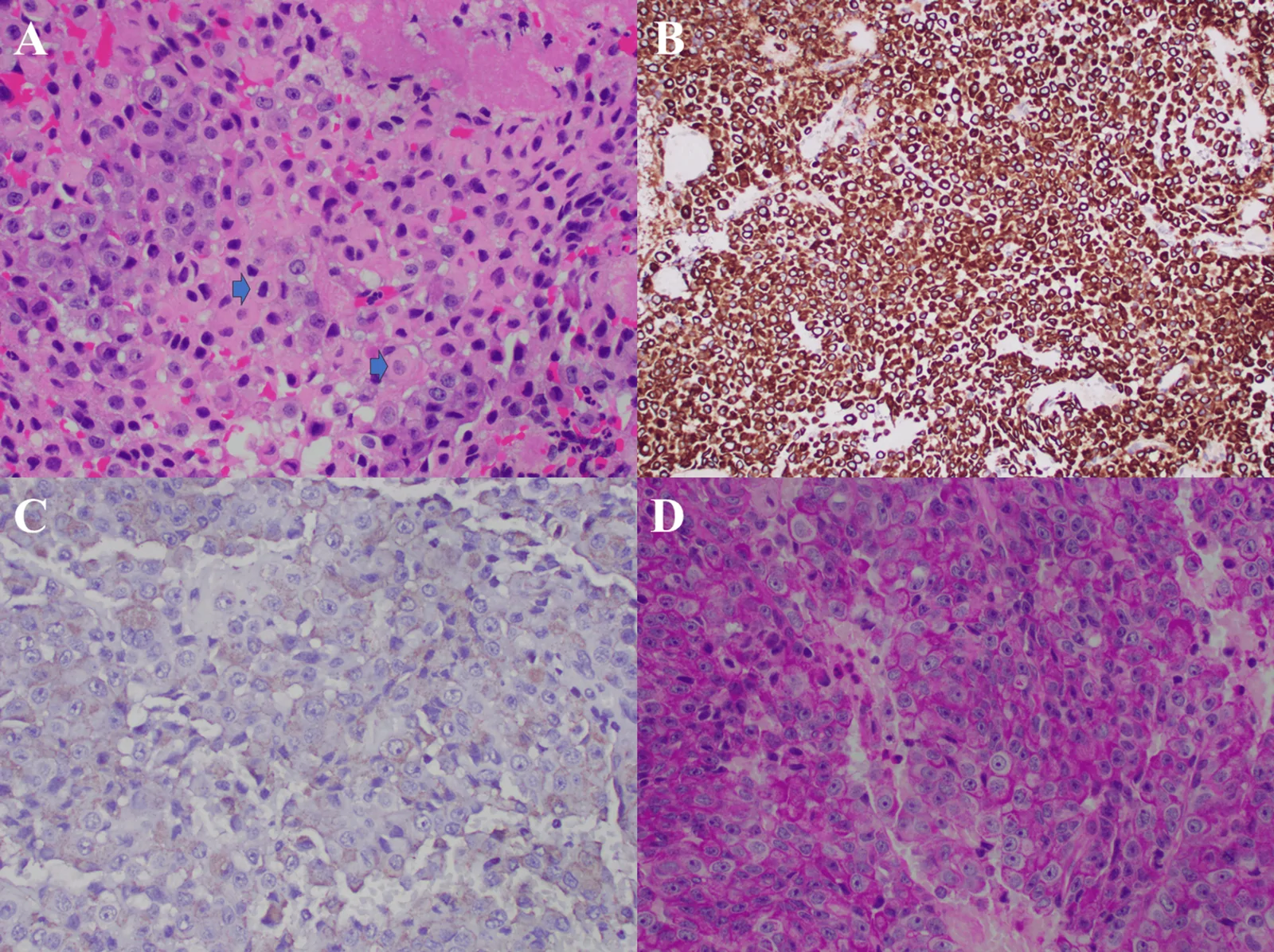
Histopathological images indicative of Crooke cell adenoma. A: Hematoxylin and eosin-stained (H&E) slide shows polygonal cells with prominent nucleoli and perinuclear hyaline material. B: Tumour cells show prominent concentric staining pattern for CAM5.2. C: Adrenocorticotropic hormone (ACTH) reactivity is dislocated to the periphery of the tumour cells. D: Perinuclear hyaline material is periodic acid-Schiff (PAS) negative.

Histopathology (Figure 4) revealed sheets of polygonal cells with prominent nucleoli and hyaline cytoplasmic change. Mitotic activity was inconspicuous. Immunohistochemistry revealed focal TPIT positivity, weak peripheral cytoplasmic ACTH and PAS staining, and prominent perinuclear and ring-like CAM5.2 expression. Marker of proliferation Kiel 67 (Ki-67) index was approximately 3%. Guanine, adenine, thymine, and adenine 3 (GATA3), growth hormone (GH), and thyroid-stimulating hormone (TSH) were negative. Reticulin staining showed disruption of the reticulin framework. All these findings were consistent with CCA. No non-neoplastic anterior pituitary tissue was available for assessment of Crooke hyaline change. This is a recognised limitation of subtotal resection in apoplexy.

His right oculomotor palsy improved immediately postoperatively and fully resolved by two weeks, with no recurrence at three-month follow-up. Right eye visual acuity improved from 6/12 pre-operatively, to 6/7.5 on day 3, to 6/6 by one month. Adrenal biomarkers had improved by the time of discharge on postoperative day six, and he remained endocrinologically silent, with normal short synacthen test and overnight dexamethasone suppression test results at one and three months (Table 1).

Given the aggressive nature of this tumour and residual disease, adjuvant volumetric modulated arc therapy (VMAT) was commenced three months postoperatively (54 Gy in 30 fractions). Repeat MRI of the brain at three months post-radiotherapy demonstrated stable residual at the cavernous segments of both ICAs.

## Discussion

An exceptionally rare presentation

CCAs account for fewer than 1% of all PitNETs [5,17], yet carry disproportionate clinical risk. George et al. identified only 36 CCAs across several decades at the Mayo Clinic, with a 60% recurrence rate, 24% multiple recurrences, and 12% disease-related mortality [4]. Findlay et al., in a multicentre analysis of 2,826 surgically treated PitNETs, confirmed significantly lower rates of hormonal normalisation (50% vs. 78%) and biochemical remission (75% vs. 84%) compared to typical corticotroph adenomas, even when gross total resection (GTR) was achieved. These findings were accompanied by a 50% recurrence rate and a 9% metastatic rate [5]. The 2022 WHO Classification formally recognises CCAs as a distinct entity with malignant potential [1], underscoring the importance of early and accurate histopathological identification.

This case is notable for the further convergence of two independently uncommon features: endocrinological silence in an aggressive ACTH-lineage tumour, and apoplectic presentation in a subtype rarely documented to haemorrhage. Together, these features obscure diagnostic certainty and delay intervention.

Endocrinological silence in Crooke cell adenoma

CCAs are T-box transcription factor (TPIT)-positive corticotroph tumours, classified by their transcription factor lineage, and are intrinsically ACTH-producing. However, 35% are endocrinologically silent without evidence of biochemical or clinical hypercortisolism [4]. The pathophysiological basis remains incompletely understood. One hypothesis proposes that glucocorticoid-driven cytokeratin accumulation mechanically displaces ACTH granules to the cell periphery, disrupting normal intracellular processing despite preserved TPIT expression [2,12]. Others argue on the contrary - irregular processing of proopiomelanocortin (POMC), the precursor of ACTH [13], and cytokeratin sequestration paradoxically disrupts the negative feedback mechanism of ACTH synthesis and secretion [4,16]. These competing models have not been resolved.

Clinically, silent CCAs remain difficult to diagnose. Corticotroph lineage was identified only on postoperative immunohistochemistry, which delayed diagnosis. This carries direct implications for surveillance too. Without a hormonal surrogate, recurrence can only be detected through serial MRI. Silent corticotroph adenomas demonstrate significantly shorter progression-free survival than other nonfunctioning PitNETs (24.5 vs. 51.1 months), underscoring the need for close, long-term neuroimaging follow-up of aggressive CCAs [18].

CCA apoplexy: underrecognised rather than truly rare

Pituitary apoplexy complicates 2%-12% of pituitary adenomas [8], predominantly nonfunctioning tumours (45%-80%) [19]. Corticotroph adenoma apoplexy is uncommon (5.7% of cases in one large surgical series) [20], and CCA apoplexy remains exceedingly rare. This is only the tenth documented instance of CCA apoplexy - the other nine cases are summarised in Table 2. Tumour biology, however, predicts a higher incidence. Muradov et al. identified apoplexy in 14.3% of CCA patients versus 1.7% of non-CCA corticotrophs, an 8.5-fold elevated risk [16]. None of the other major CCA series have specifically documented apoplexy as a presenting feature for CCAs [3-5,17]. 

**Table 2:** Summary of all documented cases of Crooke cell adenoma apoplexy ACTH: Adrenocorticotropic hormone, PAS: Periodic acid-Schiff; CCA: Crooke cell adenoma; STR: subtotal resection; Ki-67: Kiel 67 index; TSS: transsphenoidal surgery; MIB1: Mindbomb Homolog-1 Index.

Author, Year, Journal	No of cases	Clinical Findings	Biochemical Profile	Histology/ Immunohistochemistry	What is Unique?
Roncaroli F et al., 2002, Endocr Pathol [12]	2 (49F)	No Cushing disease, severe headache, diplopia	Serum ACTH, serum cortisol, 24-h urine cortisol normal; other hormones normal	Reticulin network disrupted; Cytoplasm PAS +ve; perinuclear PAS -ve; Diffuse ACTH +ve around periphery; CAM5.2 +ve; Ki67 1%.	First two documented cases of CCA presenting with apoplexy. Treated with surgery +- hormone replacement
	(59M)	No Cushing disease; tired, malaise, weak, constipation x 1y	Serum ACTH elevated; Serum cortisol, 24-h urine cortisol normal; hypothyroidism, hypogonadism	Same as above, except Ki67 0.5%	As above
Todnem N et al., 2018, World Neurosurg [13]	1 (55F)	No Cushing disease; intermittent headache and dizziness x 1m	Serum ACTH, serum cortisol, 24-h urine cortisol normal; Other hormones normal	Cytoplasm PAS +ve; Diffuse ACTH +ve; CAM 5.2 +ve; Ki67 <2%	CCA apoplexy treated with surgery (STR) only MRI at 1 year no recurrence
Khatri KJ et al., 2019, AACE Clin Case Rep [14]	1 (64M)	No Cushing disease; left ear pain; left- sided headache; tinnitus, ptosis and left oculomotor palsy	Serum ACTH, serum cortisol elevated (secondary to stress response); Serum prolactin low	ACTH +ve around periphery; Anti-cytokeratin antibody +ve with ring-like displacement of granular cytoplasm ;Ki-67 <3%; MIB1 <3%; p53 1%	Recurrent apoplexy after previous TSS (>10 years prior – likely a cell line transformation, rather than the same pathological subtype) Treated with 2^nd^ TSS and SRS (1.8Gy x 28 sessions) MRI at 3 months showed stable residual in both cavernous and sphenoid sinus
Krug RG 2nd et al., 2019, World Neurosurg [15]	1 (45M)	No Cushing disease; headache and diplopia	Serum ACTH mildly elevated; Serum cortisol normal	ACTH +ve around periphery; CAM 5.2 +ve; Ki67 rare	Unique association of apoplexy with two ICA pseudoaneurysms. Is this caused by its invasiveness? Treated with surgery to apoplexy+embolisation of pseudoaneurysms; MRI at 2 years - no recurrence
de Silva et al., 2021, BMC Endocrine Disorders [11]	1 (35F)	Cushing Disease: Refractory hypokalaemia, dorsocervical/supraclavicular fat pads, proximal weakness, acne, round face, pigmentation; bifrontal headache, blind in left eye, partial visual loss in right eye for 1 week	Serum ACTH, serum cortisol elevated; autonomous ACTH secretion	ACTH +ve around periphery; AE1/AE3 cytokeratin stain showed ring-like positivity in most cells (CAM5.2 unavailable); Ki-67 <1%; p53 3%	First reported case of CCA apoplexy in a patient with active Cushing disease. Biochemical and clinical resolution noted post-operatively. MRI at 1 year - no recurrence.
Muradov et al., 2026, Clinical Endocrinology [16]	3	No clinical details were given for the three CCA apoplexy cases specifically. Comparable % of patients with visual field defects.	CCAs had comparable biochemical profiles (serum cortisol; 24-h urine cortisol, dexamethasone suppression tests) with non-CCA corticotrophs, except serum ACTH (higher, p=0.007).	ACTH +ve; CK8/18 used as cytokeratin marker instead of CAM5.2	3/21 with CCA had apoplexy versus 1/60 with non-CCA corticotrophs. Similar post-operative biochemical remission and recurrence rates. CCA patients had larger tumour sizes, higher Ki67 indices, and residual disease.

Several mechanisms account for the elevated apoplectic risk. CCAs predominantly present as macroadenomas (80%) [4] and demonstrate higher Ki-67 proliferative indices [21] than non-CCA corticotrophs. Macroadenomas are more likely to outgrow their vascular supply, creating ischaemia foci within hypovascular parenchyma [22]. Higher Ki-67 proliferative index implies rapid cellular turnover, predisposing to spontaneous infarction [22]. Cavernous sinus invasion (46-72% of CCAs) may compromise ICA integrity directly [3,4]. Krug et al. described an apoplectic silent CCA associated with two ICA pseudoaneurysms, attributed to direct tumour erosion [15]. Subclinical haemorrhage is present in up to 17% of resected pituitary tumours and largely goes unreported [10]. CCA apoplexy is therefore more likely underrecognised than truly absent.

CCA apoplexy carries greater danger than apoplexy of other pituitary tumour subtypes. The high rate of cavernous invasion [3,4] predisposes patients to acute cranial neuropathy. In this patient, a partial right oculomotor palsy progressed to a complete palsy with fixed mydriasis in under 24 hours, mandating urgent surgical decompression. This rapid deterioration has been reported in both de Silva et al. [11] and Khatri et al [14]. Bilateral ICA encasement in this patient precluded complete resection and limits the feasibility of reoperation, radiation therapy [17,23] and temozolomide therapy [7,21,24-26]. Complete oncological control can be difficult to achieve when critical vessels are involved.

Histological diagnosis: barriers and recommendations

Accurate histological subtyping of apoplectic specimens is constrained by specimen quality. In Giritharan et al.'s series, 60% of specimens contained predominantly necrotic tissue and 76% showed haemorrhage, necrosis, or both [27], limiting immunohistochemical yield. Sampling bias compounds this: tissue retrieved from haemorrhagic or necrotic zones may not represent the viable periphery where Crooke cell morphology is best preserved. In this case, definitive CCA diagnosis was achieved despite apoplectic haemorrhage and subtotal resection limiting the amount of viable tissue obtained, confirming that accurate subtyping is feasible in apoplexy when adequate non-necrotic tissue is sampled.

The presence of Crooke hyaline change in peritumoral non-neoplastic corticotropic tissue would have provided additional histological corroboration on the diagnosis of CCA. Crooke hyaline change is observed in 74%-83% of functioning corticotroph tumours [28,29] but is consistently absent in silent corticotroph adenomas and pseudo-Cushing states [30-32]. In this case, no non-neoplastic anterior pituitary tissue was available for assessment, further illustrating how apoplexy and subtotal resection can compound diagnostic ability.

The WHO classification provides clear criteria for corticotroph subtyping, based on TPIT expression, ACTH immunoreactivity, and cytokeratin deposition patterns [1]. Most centres follow a two-step panel (transcription factors, followed by hormonal immunohistochemistry). However, there are currently no standardised protocols for apoplectic specimens, introducing diagnostic inconsistency between institutions. Sood et al. found similar Allred scores between TPIT and ACTH in detecting corticotroph PitNETs and concluded that both are interchangeable depending on laboratory availability [33]. However, McDonald et al. demonstrated superior sensitivity and specificity for TPIT over ACTH immunostaining, especially in silent tumours with weak or absent ACTH expression [34]. In resource-constrained settings, AE1/AE3 [11] and CK8/18 [16] may even substitute CAM5.2 for cytokeratin testing, even though CAM5.2 is more specific to CCA. Kober et al. found that CCAs were significantly more likely to have TP53 mutations than other corticotroph tumours [6]. We advocate for the development of a standardised minimum staining panel for all apoplectic corticotroph specimens, consistent with the WHO classification [1].

Adjuvant radiotherapy and surveillance

Urgent surgical decompression is the cornerstone of management in CCA apoplexy with neurological compromise. The endoscopic endonasal transsphenoidal approach provided adequate access for tumour debulking and chiasmal decompression without the morbidity of transcranial surgery. Subtotal resection was appropriate and intentional in this case given bilateral ICA encasement: the vascular risk of radical resection outweighs the benefit when adjuvant radiotherapy is available. Satisfactory chiasmal decompression and neurological recovery were achieved. There is currently no standardised adjuvant protocol specific to CCA. While Snyder et al. reported tumour control in all five patients treated with gamma knife radiosurgery (median dose 25 Gy), endocrine remission was achieved in only 60% [23]. Giraldi et al. reported good outcomes with fractionated radiotherapy in one CCA with residual disease [17]. Amongst CCA apoplexy cases (Table 2), almost all were managed with surgery alone, with only one undergoing stereotactic radiosurgery on the second resection for recurrent disease [14]. This patient is the first to demonstrate the efficacy of adjuvant VMAT in preventing recurrence of this aggressive tumour that is more likely to have residual disease post-operatively. He tolerated radiotherapy well and had stable residual disease at three months.

Unlike functioning CCAs where serum ACTH and urinary free cortisol serve as sensitive markers of recurrence, post-treatment surveillance of silent CCAs relies exclusively on serial MRI. There are no guidelines that define optimal MRI intervals for post-apoplexy CCA. Given the aggressive biological profile, we recommend MRI surveillance every three to six months for the first two years, then annually if stable.

Limitations

The rarity of CCA apoplexy precludes definitive conclusions regarding the relationship between CCA histology and apoplexy risk. Current evidence is limited to case reports and one comparative series. Absence of routine histopathological subtyping in apoplexy series likely means further cases go undetected. Multicentre registries with standardised histopathological classification [5] are needed to characterise the natural history, optimal surveillance frequency, and long-term outcomes of this understudied population.

## Conclusions

This case is the tenth documented instance of CCA presenting with pituitary apoplexy, and adds to the small body of evidence on endocrinologically silent CCA apoplexy. The paucity of reported cases reflects a stark underdiagnosis of an otherwise aggressive tumour, driven by its often silent presentation, absence of standardised histological protocols, and immunohistochemical constraints in necrotic tissue. Four conclusions emerge. First, CCA must be actively excluded in corticotroph PitNETs presenting with apoplexy. Viable tissue should undergo a minimum staining panel. Second, endocrinological silence must not be equated with benign behaviour; it is falsely reassuring. Tumour aggressiveness is determined by morphology and molecular profile, not cortisol levels. Third, this case is the first to demonstrate that subtotal resection with adjuvant fractionated radiotherapy is safe and effective in CCA apoplexy complicated by ICA encasement. Fourth, post-treatment surveillance in silent CCAs requires serial MRI given the absence of biochemical markers. Given the diagnostic challenges of endocrinologically silent adenomas, multicentre collaboration and prospective registry data are essential to establish evidence-based surveillance and treatment protocols for this rare but clinically consequential entity.
